# Scrotal Circumference and Its Relationship with Testicular Growth, Age, and Body Weight in Tho Tho (*Bos indicus*) Bulls

**DOI:** 10.1155/2014/249537

**Published:** 2014-10-29

**Authors:** P. Perumal

**Affiliations:** Animal Reproduction Laboratory, National Research Centre on Mithun (ICAR), Jharnapani, Nagaland 797 106, India

## Abstract

The present study was undertaken to assess the relationship between the scrotal circumference and testicular parameters with body weight and age in Tho Tho bulls (*Bos indicus*), which were maintained at around the villages of National Research Centre on Mithun (ICAR), Jharnapani, Nagaland, India. A total of 32 Tho Tho bulls were selected and divided into four groups according to their age and each group consisted of 8 bulls, namely, Group I: 18–24 months (*n* = 8), Group II: 25–36 months (n = 8), Group III: 37–48 months (*n* = 8), and Group IV: 49 months and above (*n* = 8). The scrotal circumference and testicular parameters were measured with caliper and tape and age of animals was calculated with dental formula. The body weight of bulls was estimated with Shaeffer's formula. Result revealed that the scrotal circumference was highly correlated with testicular parameters and body weight compared to age. Compared to exotic cattle (*Bos taurus*), Tho Tho bull's testicular parameters and scrotal circumference were lower. The results of the present study in Tho Tho bulls revealed that scrotal circumference is a useful indicator and is an important selection criterion to determine the testicular development and breeding soundness in young bulls as it is highly correlated with tesicular parameters.

## 1. Introduction 

Tho Tho cattle (*Bos indicus*) is a local indigenous bovine species of North Eastern Hilly (NEH) region especially in Nagaland state of India. It is rearing under semi-intensive system. The bull is half of the herd in animal husbandry, which indicates that the sire is one of the parents of all the calves in the herd. Since the bull has more genetic influence (80–90%) of calves it sires, fertile bull selection can be the most powerful method for improvement of the herd. The demand for sperm from outstanding sires has increased with the development of frozen semen technology and the growth of large artificial breeding organizations. Methods to predict sperm production potential and particularly to identify the bulls with high sperm output potential at an early age are important, which is related with testicular parameters and scrotal circumference. Traditionally, Tho Tho bulls have been selected on the basis of growth, rather than on reproductive traits. However, reproduction is one of the most important factors for the economics of livestock production. Generally, testicular measurements at early ages are very useful in selection of breeding sire to collect semen for artificial insemination or breeding purpose. The most important parameters are testicular diameter, testicular length, testicular volume, and scrotal circumference. The males with bigger testes produce more sperm than the males with smaller testes [1]. Regression equations have revealed that testicular size was positively related to body weight and age [2]. Positive correlations between prepubertal male hormone levels and subsequent testes size and mating frequency have also been reported [3]. These are the potentially useful indicators of reproductive traits and have been used to compose selection indices for Tho Tho bulls to use in breeding purpose. These traits are easily measured and correlated with body weight and reproductive performance [4–6]. Testicular weight and body growth of large number of dairy bulls were measured to develop techniques which would give repeatable results and to determine which one was most highly correlated with scrotal circumference. This study also was designed to provide the basis for investigation of parameters useful in predicting future reproductive performance and in establishing the extent to which differences in these parameters are varied.

Measurement of scrotal circumference and testicular parameters is an essential part of breeding soundness evaluation of breeding bulls. Measurement of these parameters especially scrotal circumference has a great value on onset of puberty, total semen production, semen quality, pathological conditions of reproductive system, and the fertility or infertility status of breeding bulls [7]. Moreover, testicular measurements have been utilized as the indicators for reproductive capabilities in the postpubertal period of bulls [8]. Scrotal circumference is highly correlated with body weight and age [9] and it is highly correlated with testis weight [10] and testicular consistency is correlated with fertility [11]. Scrotal circumference and testicular parameters have a direct relationship with sperm production. Testis size and body weight provides idea about the physical and physiological maturity of the bulls, its sperm output, and birth weight of its young ones [12]. There are various reports on the testicular growth and related to semen attributes in matured dairy bulls [13–18], buffaloes [19–21], ram [22], and mithun bulls [23]. If these reproductive traits are to be incorporated into management and breeding programs, it is desirable that their relationships to other traits are to be understood. There are various factors involved in influencing scrotal circumference and testicular measurements such as age, breed of the bulls [24], and season of the year [25].

Studies associating with body weight, testicular measurements, and scrotal circumference obtained during the different phases of growth of Tho Tho bovine species are scarce. In this respect, it is not only necessary to determine how these traits respond to selection at different ages, but also to determine the magnitude of correlation between these traits. Therefore, the present study was designed to estimate the testicular parameters at different ages and correlate with body weight and age in Tho Tho bulls to provide data for the definite selection of Tho Tho bulls for future breeding purpose and conservation of this precious indigenous bovine species.

## 2. Materials and Methods

### 2.1. Experimental Animals

A total of thirty-two apparently healthy Tho Tho bulls with different age groups were selected from the herd of villages at around the National Research Centre on Mithun (ICAR), Nagaland, India. The study area lies between 25°54′30′′ North latitude and 93°44′15′′ East longitude and at an altitude range of 250–300 mean sea level. The animals were maintained under semi-intensive condition of grazing in the forest during day time and ting them in shed during night time. The experimental animals in shed were fed daily with* ad libitum* quantity of locally available forages without providing concentrate. Fresh water was available throughout the day.

Age of the bulls was determined using their dental formula. The body weight of each bull was calculated according to Shaeffer's formula [25]: *L* × *G* ^2^/300 × 2.2, where *L* = body length in inch and *G* = girth in inch. According to their age, the experimental bulls were equally divided into four groups, each of 8 bulls, namely, Group I: 18–24 months (*n* = 8), Group II: 25–36 months (*n* = 8), Group III: 37–48 months (*n* = 8), and Group IV: 49 months and above (*n* = 8).

### 2.2. Testicular Measurement

Testicular parameters and scrotal circumference were measured with a caliper (Mitutoya Digimatic Caliper) and a tape after restraining the bull in the chute [26]. Testicular length was measured by placing the fixed arm of the caliper at the proximal end and the sliding arm at the distal end of the testes. Care was taken to exclude the epididymides. Thickness or depth was measured by placing the fixed arm of the caliper at the anterior aspect and the sliding arm at the posterior aspect of the each testis, at the point of maximum depth. Width of each testis was measured by sliding the other testes up in the scrotum and placing one arm of the caliper at the medial aspect and the other at the lateral aspect, at the point of maximum width. For measurement of scrotal circumference, the testicle was pushed firmly into the bottom of the scrotum by placing the thumb and fingers laterally on the side of the neck of the scrotum and pushed ventrally. A flexible cloth tape was formed into a loop and slipped over the scrotum and scrotal circumference was measured in centimeters by pulling the tape snugly around its greatest diameter [26]. Testes volume was calculated by using the formula for volume of an ellipsoid, that is, 4/3*πabc*, where *a* = thickness/2, *b* = width/2, and *c* = length/2 (Love et al. 1991). Weight of the testes was measured by multiplying volume with 1.038 which is the approximate density of testicular tissue in cattle [27].

### 2.3. Statistical Analyses

The results were analysed statistically and expressed as the mean ± S.E.M. Means were analyzed by one way analysis of variance between the different age groups of Tho Tho bulls, followed by Tukey's post hoc test to determine significant differences between the groups using the SPSS/PC computer program (version 15.0; SPSS, Chicago, IL). Differences with values of *P* < 0.05 were considered to be statistically significant. Correlation coefficient was estimated among the testicular parameters, scrotal circumference, age, and body weight of the bulls. Differences at *P* < 0.05 and *P* < 0.01 were considered to be statistically significant.

## 3. Results 

**Figure 1:**
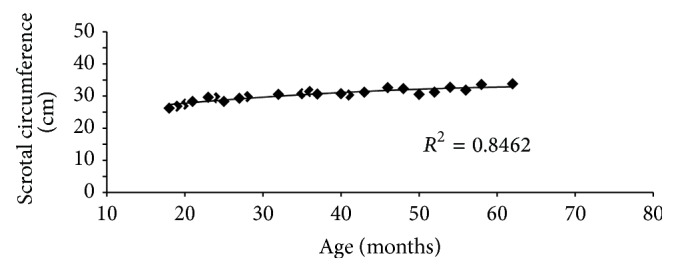
Relationship between scrotal circumference and age in Tho Tho (*Bos indicus*) bulls.

**Figure 2:**
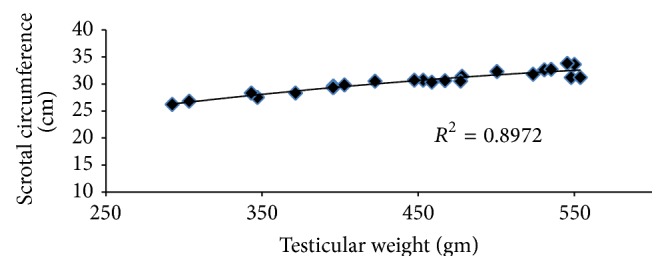
Relationship between scrotal circumference and testis weight in Tho Tho (*Bos indicus*) bulls.

**Figure 3:**
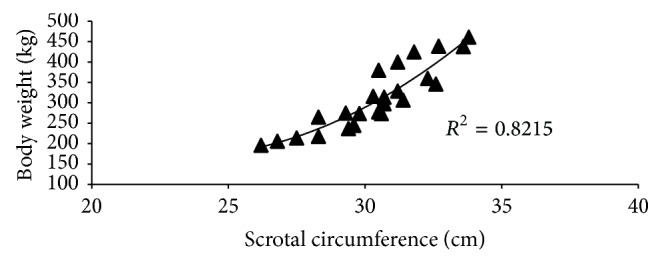
Relationship between scrotal circumference and body weight in Tho Tho (*Bos indicus*) bulls.

The results of different testicular parameters, scrotal circumference, and body weight at different age of Tho Tho bulls were presented in Table 1, which indicated that these parameters were significantly (*P* < 0.05) different between the age groups. It has been reported that the average estimated testicular weight was 18.50, 40.20, and 51.25% greater in bulls aged 25–36, 37–48, and >49 months of age than in 18–24-month-old bulls. Similarly the average estimated testicular volume was 15.50, 28.69, and 33.85% greater in bulls aged 25–36, 37–48, and >49 months of age than in 18–24-month-old bulls. In case of scrotal circumference, the average estimated value was 6.76, 10.58, and 13.35% greater in bulls aged 25–36, 37–48, and >49 months of age than in 18–24-month-old bulls. Correlations between the different measurements for the 32 bulls are shown in Table 2. Similarly as expected, various measurements of the testis were highly correlated with each other (*P* < 0.01) as in exotic cattle [18], buffalo [21], and mithun [23]. Body weight was more highly correlated with the various testicular measurements than age. But also according to age, the testicular parameters were increased significantly (*P* < 0.05) in Tho Tho cattle bulls. Moreover age (Figure 1), testis weight (Figure 2), and body weight (Figure 3) of Tho Tho cattle bulls were highly correlated with scrotal circumference. Scrotal circumference was highly correlated with other testicular measurements as well as testicular volume. Scrotal circumference was a simple measurement to obtain; maximum circumference is reached at 5-6 years of age and remains relatively constant thereafter. These results indicate that scrotal circumference is a useful indicator and is an important selection criterion to determine the testicular development in young bulls and it has highly correlation with testicular parameters.

## 4. Discussion

In the present study, the results revealed that scrotal circumference of Tho Tho bulls have been highly correlated with other testicular parameters. Thus, measurement of scrotal circumference in Tho Tho bulls is very useful to predict the testicular parameters and can be used in breeding centre to select suitable breeding male for artificial breeding purpose.

There was no report on effect of scrotal circumference on testicular parameters in Tho Tho cattle and, to the best of our knowledge, this is the first report on the effect of scrotal circumference on testicular parameters in Tho Tho bulls. Analysis of various testicular parameters such as testis length, width, thickness, volume, and weight is important for extensive selection of Tho Tho bulls for future breeding and conservation program in the NEH region especially in Nagaland state.

Correlation coefficients measured among the different testicular measurements and scrotal circumference for the 32 bulls were presented in Table 2. Body weight was somewhat more highly correlated with the various testicular measurements than with age. As would be expected the various measurements of testis size were highly correlated with each other. All correlation coefficients were highly significant (*P* < 0.01). Circumference of the testes was highly correlated with other testicular measurements (Table 2); it was highly repeatable (Table 1) and it was a simple measurement to obtain and easy to interpret. The correlations among this trait and other testicular measurements on a within-age group basis are not given, but they remained high. The correlation of 0.94 between scrotal circumference and testis weight is similar to the values of 0.90 to 0.96 reported by other researchers [28] for various comparisons between testicular dimensions and weight. Since circumference of the testes was easy to measure, the measurement had high repeatability and it was highly correlated with testis weight and volume and this measurement subsequently was taken on all bulls examined.

In the present study, the correlation among the various testicular measurements were in agreement with the study in Nigerian indigenous bulls [29], Bangladesh indigenous bulls [30], Sahiwal bulls [28], buffalo [19], and Mithun bulls [23], but are at variance with another report on other bulls in which testes volume and length were not correlated with age and body weight [20]. In our experience, Tho Tho bulls are indigenous animals, maintained under semi-intensive system and were sensitive to handle the testes and other reproductive organs. So that it was difficult to take measurement accurately. Also in the earlier study of other cattle bulls, testicular volume was calculated by multiplying mean values for length, thickness, and total width of the testes [19]. In our experiment, in this procedure, results were in higher side than those obtained by using the formula for estimating the volume of an ellipsoid which has a very high accuracy [31].

Interestingly, in the present study, within each age group, the testicular measurement of Tho Tho bulls were lower than those reported for Holstein bulls and higher than buffalo bulls [21], but maximum scrotal circumferences was reached at nearly the same age [18]. Nevertheless, there appears to be ample scope for selecting Tho Tho bulls with large scrotal circumference since about 60 to 70% of bulls, within each age group had a scrotal circumference greater than the mean scrotal circumference for that age group. In young dairy and beef bulls, scrotal circumference and testes size were highly correlated [18, 32] and bulls designated questionable or unsatisfactory potential breeders have smaller scrotal circumference measurements [7]. But the reports in other species like bubaline [21] and bovine [16] species showed that the spermatogenesis was higher in younger than older age. So that further studies with semen collection and more number of animals will help to pinpoint the exact age at which the mature rate of spermatogenesis is attained.

In the present study, the mean value of scrotal circumference in Group II (25–36 months) was significantly (*P* < 0.05) higher than Group I (18–24 months) and Group IV higher value than the other three groups. It has been observed in the present study that SC was increased rapidly in young bulls and gradually in mature bulls. But it has been reported that in old age this parameter is decreased due to senile atrophy [33]. In the present study also, the proportion of increasing the scrotal circumference and testicular parameters was low in older bulls than in growing younger bulls. Based on our results on the mean testicular circumference of 32 Tho Tho bulls, we recommend that, for optimum breeding potential and possible higher fertility, Tho Tho cattle bulls with a mean scrotal circumference of ≥27.97, 30.00, 31.28, and 32.27 cm in the age groups of 18–24, 25–36, 37–48, and >48 months, respectively, should be selected as the breeding sires.

Scrotal circumference (Table 1) was increased with age and following a pattern similar with BW (Figure 1), but testes tended to reach mature size more rapidly, as indicated by the linear relationship between SC and BW (Figure 2). This is consistent with an earlier report [24].

High correlation was observed between testis size and scrotal circumference and based on this collective information, testicular diameter along with scrotal circumference are excellent indicators of spermatogenic function [33] and this can be used for breeding soundness evaluation in Tho Tho bulls [34]. In conclusion, present results indicate that as in exotic cattle [18] and buffalo [21] and Mithun [23] bulls, scrotal circumference in the Tho Tho bulls is a useful indicator of breeding soundness and should be used as an important criterion for selection of young bulls for breeding purpose. However, estimates of changes in testicular development can be considerably improved by following the same bulls at different ages to avoid confounding between bulls and ages. Future, semen collection and the seminal parameters will be correlated with testicular and scrotal parameters are warranted to confirm the present findings.

## Figures and Tables

**Table 1:** Scrotal circumference, testicular, and seminal parameters of Tho Tho (*Bos indicus*) bulls at different ages (mean ± S.E.).

Scrotal and testicular characters	18–24 months (*n* = 8)	25–36 months (*n* = 8)	37–48 months (*n* = 8)	49 months and above (*n* = 8)
Age	20.83 ± 1.52^a^	30.50 ± 2.12^b^	42.50 ± 2.24^c^	55.33 ± 2.08^d^
Body weight (Kg)	219.33 ± 4.31^a^	282.83 ± 4.01^b^	323.17 ± 5.47^c^	423.83 ± 5.56^d^
Scrotal circumference (cm)	27.97 ± 1.18^a^	30.00 ± 1.05^b^	31.28 ± 0.98^bc^	32.27 ± 1.22^c^
Left testes length (cm)	8.84 ± 0.68^a^	9.63 ± 0.59^b^	9.63 ± 0.66^b^	9.78 ± 0.60^b^
Left testes width (cm)	5.72 ± 0.52^a^	5.77 ± 0.54^a^	6.58 ± 0.44^b^	6.82 ± 0.38^b^
Left testes thickness (cm)	5.88 ± 0.61^a^	6.23 ± 0.38^b^	6.45 ± 0.37^bc^	6.87 ± 0.40^c^
Left testes volume (cm^3^)	146.53 ± 4.12^a^	181.46 ± 4.68^b^	213.80 ± 4.23^c^	239.05 ± 4.64^d^
Left testes estimated weight (gm)	152.09 ± 4.20^a^	188.35 ± 4.74^b^	221.93 ± 4.20^c^	248.13 ± 4.38^d^
Right testes length (cm)	9.74 ± 0.69^a^	10.03 ± 0.61^ab^	10.40 ± 0.77^b^	10.25 ± 0.60^ab^
Right testes width (cm)	6.11 ± 0.52^a^	6.26 ± 0.49^a^	6.99 ± 0.41^b^	7.19 ± 0.37^b^
Right testes thickness (cm)	6.21 ± 0.48^a^	6.67 ± 0.50^b^	6.85 ± 0.53^bc^	7.12 ± 0.43^c^
Right testes volume (cm^3^)	191.66 ± 5.78^a^	219.25 ± 4.56^a^	260.37 ± 5.26^b^	272.48 ± 4.12^b^
Right testes estimated weight (gm)	198.95 ± 5.42^a^	227.58 ± 4.98^a^	270.26 ± 5.09^b^	282.83 ± 4.20^b^
Total volume (cm^3^)	338.19 ± 6.59^a^	400.71 ± 6.75^b^	474.17 ± 6.29^c^	511.53 ± 5.24^c^
Total weight (gm)	351.04 ± 6.71^a^	415.94 ± 6.88^b^	492.19 ± 6.40^c^	530.97 ± 5.32^c^

Figures in parenthesis indicate number (*n*) of Tho Tho cattle bulls.

Figures with same superscript (a, b, c, and d) do not differ significantly in rows.

**Table 2:** Correlation coefficients between scrotal circumferences, age, body weight, and various testicular measurements in Tho Tho (*Bos indicus*) bulls.

Number	Measurement	2	3	4	5	6	7	8	9	10	11	12	13	14	15
1	Age	0.82^**^	0.90^**^	0.56^**^	0.91^**^	0.86^**^	0.88^**^	0.88^**^	0.62^**^	0.92^**^	0.90^**^	0.94^**^	0.94^**^	0.93^**^	0.93^**^
2	Body weight		0.89^**^	0.52^**^	0.86^**^	0.85^**^	0.85^**^	0.85^**^	0.62^**^	0.87^**^	0.88^**^	0.91^**^	0.91^**^	0.89^**^	0.98^**^
3	Scrotal circumference			0.70^**^	0.88^**^	0.93^**^	0.93^**^	0.93^**^	0.70^**^	0.86^**^	0.92^**^	0.93^**^	0.93^**^	0.94^**^	0.94^**^
4	Right testes length				0.61^**^	0.72^**^	0.82^**^	0.82^**^	0.72^**^	0.54^**^	0.64^**^	0.68^**^	0.67^**^	0.76^**^	0.76^**^
5	Right testes width					0.86^**^	0.93^**^	0.93^**^	0.57^**^	0.95^**^	0.86^**^	0.92^**^	0.92^**^	0.94^**^	0.94^**^
6	Right testes thickness						0.95^**^	0.96^**^	0.77^**^	0.83^**^	0.92^**^	0.93^**^	0.93^**^	0.96^**^	0.95^**^
7	Right testes volume							0.98^**^	0.75^**^	0.88^**^	0.88^**^	0.95^**^	0.93^**^	0.97^**^	0.96^**^
8	Right testes weight								0.76^**^	0.87^**^	0.89^**^	0.94^**^	0.94^**^	0.98^**^	0.94^**^
9	Left testes length									0.55^**^	0.64^**^	0.76^**^	0.76^**^	0.76^**^	0.76^**^
10	Left testes width										0.87^**^	0.95^**^	0.93^**^	0.92^**^	0.93^**^
11	Left testes thickness											0.94^**^	0.95^**^	0.93^**^	0.93^**^
12	Left testes volume												0.98^**^	0.98^**^	0.96^**^
13	Left testes weight													0.99^**^	0.97^**^
14	Total testes volume														0.98^**^
15	Total testes weight														1.00

^**^Correlation coefficient was highly significant, *P* < 0.01.
